# Insect SVWC Proteins: A Diverse Cytokine-like Family Orchestrating Multilayered Antiviral and Antibacterial Immunity

**DOI:** 10.3390/insects17040438

**Published:** 2026-04-20

**Authors:** Yangyang Chen, Gaoying Xu, Jingao Wang, Cong Zhang, Aliyu Yusuf Abubakar, Hengchuan Xia

**Affiliations:** School of Life Sciences, Jiangsu University, Zhenjiang 212013, China; chenyangyang232913@163.com (Y.C.); xugaoying1104@163.com (G.X.); 17787643689@163.com (J.W.); zhangcong968@163.com (C.Z.); aleeu16@gmail.com (A.Y.A.)

**Keywords:** SVWC, Vago, insect immunity, cytokine diversity, JAK/STAT pathway, pattern recognition receptor, Dicer-2, functional specialization

## Abstract

The single von Willebrand factor C (SVWC) domain-containing protein family represents a crucial class of immune molecules recently identified in insects and crustaceans. Initially regarded as functional analogs of vertebrate interferons (IFNs) due to their virus-induced expression and activation of the Janus kinase-signal transducer and activator of the transcription (JAK-STAT) pathway, recent studies have revealed that SVWC proteins possess far more complex functions. Many SVWC members are themselves a novel class of pattern recognition receptors (PRRs) that can directly bind to viruses and bacteria. Importantly, SVWCs are not a single entity but a highly diverse family—multiple subtypes exist in *Drosophila*, *Bombyx mori*, and shrimp—a gene expansion that implies functional differentiation. This review systematically examines the multifunctionality of SVWC proteins in insects and crustaceans, with a particular focus on the functional specialization driven by subtype diversity. We delve into the complex regulatory networks governing SVWC expression, including the differential activation by nuclear factor kappa B (NF-κB) pathways (Dorsal, Rel-2, Relish) and interferon regulatory factor (IRF) pathways. We detail the unique signaling mechanism by which SVWCs activate the JAK-STAT pathway via integrins, rather than the canonical Domeless receptor. Furthermore, we extend the discussion to the emerging roles of SVWCs as PRRs in humoral immunity (activating Toll/IMD pathways to induce antimicrobial peptides) and cellular immunity (mediating hemocyte phagocytosis). Based on current evidence, We propose that diverse SVWC subtypes may recognize distinct pathogens, bind to different integrin receptors, and activate specific STAT variants via disparate upstream induction pathways, thereby establishing a systematic and hierarchical immunoregulatory network. This understanding positions the SVWC protein family as a central hub in the insect immune network and offers a novel perspective on the complexity and evolution of invertebrate immunity.

## Simple Summary

The recently identified single von Willebrand factor C (SVWC) protein family plays a central role in insect immunity. This review synthesizes the diversity and multifaceted functions of the SVWC family. The SVWC family comprises numerous subtypes; some act as signaling molecules to activate immune pathways, others function as pattern recognition receptors (PRRs) to detect bacteria and viruses, and some possess dual functionality. SVWC expression is regulated by both nuclear factor kappa B (NF-κB) and interferon regulatory factor (IRF) pathways, and these proteins activate the Janus kinase-signal transducer and activator of transcription (JAK-STAT) pathway via integrins, representing a unique signaling mechanism. As PRRs, SVWCs not only induce antimicrobial peptide production but also promote phagocytosis of pathogens by hemocytes. Based on these findings, we propose that SVWC constitutes a systematic complex immunoregulatory network. This perspective not only deepens our understanding of insect immunity but also provides new theoretical foundations and potential application targets for disease control in economically important animals such as honeybees, silkworms, and shrimp.

## 1. Introduction: A Diverse Family of Cytokine-like Proteins

Innate immunity is the ancient defense line of multicellular organisms. Invertebrates, including insects, rely entirely on this sophisticated system to distinguish “self” from “non-self,” with pattern recognition receptors (PRRs) recognizing pathogen-associated molecular patterns (PAMPs) at its core.

**Figure 1:**
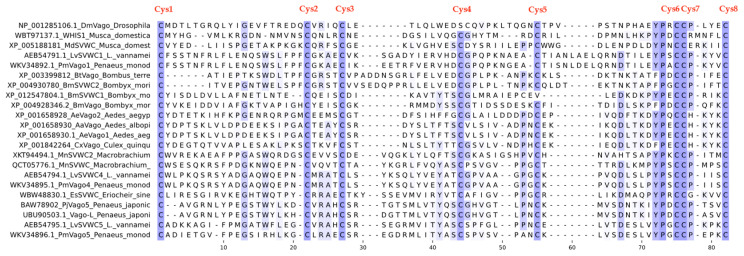
Sequence alignment of the SVWC domains from different isoforms reported in invertebrates to date. Alignment software: MEGA12; alignment method: Clustal. Highlighted residues indicate cysteine (Cys) residues. The alignment shows that the SVWC domain is highly conserved and consistently contains eight Cys residues.

**Figure 2:**
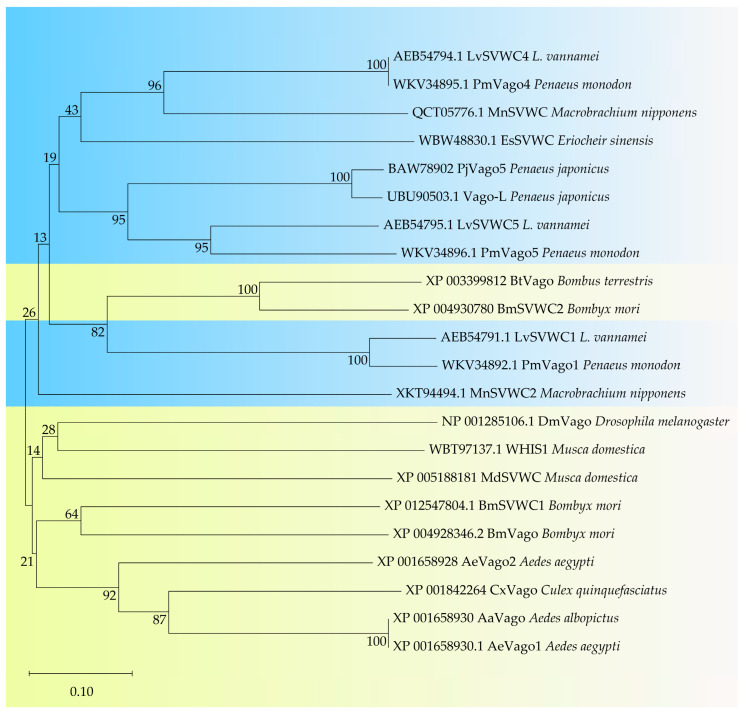
Phylogenetic tree analysis. The tree was constructed using the Neighbor-Joining method. Insects are indicated by a green background, and crustaceans by a blue background.

Within this framework, the discovery of the single von Willebrand factor C (SVWC) domain-containing protein family has opened a new window into our understanding of insect immunity. First identified in *Drosophila* in 2005 (Vago was initially used in Drosophila, and later referred to as SVWC in shrimp and silkworm, with a species prefix) [1], these proteins are characterized by a compact domain containing eight conserved cysteine residues that form four disulfide bonds essential for protein function [2,3]. Subsequently, members of this family have been widely identified in arthropods, including mosquitoes, silkworms, ticks, and shrimp [4,5,6]. Reported sequences of different SVWC subtypes are highly conserved, all containing eight cysteines (Figure 1). Previously, Labropoulou et al. had already discussed in detail the discovery and function of SVWC polypeptides from insects to crustaceans and chelicerates [7]. Gradually, a key fact has emerged: SVWC proteins are not a single entity but a highly diversified family. The *Drosophila* genome encodes multiple SVWC members; at least five *Lv*SVWC subtypes have been identified in shrimp [5], and multiple homologs exist in *Bombyx mori* [8]. This gene expansion strongly suggests that distinct SVWC subtypes have evolved unique functional roles. Moreover, the SVWC domain homology tree reveals that the SVWC protein family in invertebrates splits into two principal clades—one comprising crustaceans and the other insects—with repeated gene duplication events within each group generating multiple subtypes that may have undergone functional specialization (Figure 2).

Initially, SVWC proteins were considered functional analogs of vertebrate interferons (IFNs) due to their significant induction upon viral infection and their ability to activate the Janus kinase-signal transducer and activator of transcription (JAK-STAT) pathway, a function exhibiting conservation—for instance, both *Aa*Vago and *Cx*Vago can suppress West Nile virus (WNV) replication in *Culex* mosquitoes [2,9,10]. Despite lacking any sequence homology, the convergent evolution of activating the JAK-STAT pathway is itself highly compelling. However, recent findings have refined this simplified view: many SVWC members are themselves a novel class of PRRs capable of directly binding viruses, bacteria, and fungi [8,11,12]. This suggests that SVWC proteins may possess a dual identity, functioning as both “sensor” and “signal transducer,” thereby positioning them as a central hub in the insect immune network.

More importantly, SVWC diversity implies a complex, hierarchical immunoregulatory network. Just as vertebrates possess multiple IFNs (IFN-α, IFN-β, IFN-γ) and interleukins (ILs) (e.g., IL-2, IL-6, IL-10) that activate different JAK-STAT combinations (JAK1/JAK2/TYK2 with STAT1/STAT2/STAT3, etc.) to regulate distinct immune responses, invertebrate SVWC subtypes may similarly play a “multi-instrument” role. They may be induced by different PRR pathways, recognize different pathogens, bind different receptors (including various integrin subtypes), activate different JAK and STAT variants, and ultimately initiate tailored transcriptional programs.

While research on insect antiviral immunity is relatively mature, recent discoveries have significantly enriched this field. These advances include the recognition of *Bombyx mori* nucleopolyhedrovirus (BmNPV) p53 protein by voltage-dependent anion channel 2 (VDAC2) [13] and antiviral responses mediated by uric acid metabolism [14], reactive oxygen species (ROS) [15], and pattern recognition receptors [16]. In contrast, the mechanisms by which insect SVWC proteins participate in antiviral defense have only recently garnered significant attention. This review aims to systematically synthesize and critically discuss the multifunctionality of insect SVWC proteins, specifically focusing on the functional differentiation arising from their subtype diversity. We will explore the intricate regulatory networks governing their expression, examine the unique mechanism by which they activate the JAK-STAT pathway, and extend the discussion to their emerging roles in antibacterial defense. By integrating research from model insects like *Drosophila*, mosquitoes, silkworms, and crustaceans like shrimp, we aim to depict a comprehensive portrait of SVWC proteins as central “multi-taskers” in the insect immune system and uncover their profound evolutionary and functional significance.

## 2. SVWC Subtype Diversity and Functional Differentiation

**Figure 3:**
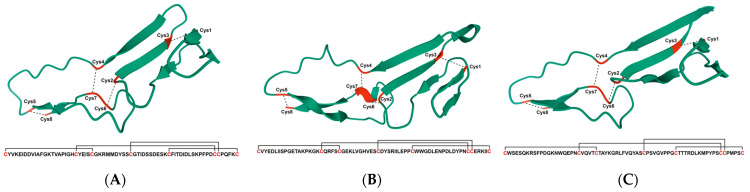
Three-dimensional structural diagrams of SVWC domains. (**A**) BmSVWC3 (functions solely as a signaling molecule). (**B**) MdSVWC (functions solely as a pattern recognition receptor). (**C**) MnSVWC (functions both as a signaling molecule and a pattern recognition receptor). The residues highlighted in red are cysteine (Cys), and the dashed lines represent disulfide bonds.

Currently reported SVWC subtypes exhibit specific inhibitory effects against distinct viruses and operate via different activation mechanisms (Table 1). However, recent findings have gradually unveiled a new possibility: SVWC proteins may possess functional duality. They can act both as cytokine-like signaling molecules and as PRRs, with some subtypes even embodying both identities simultaneously (Figure 3). This dual identity appears to have diverged among different subtypes. Although supporting evidence for this hypothesis remains limited at present—having been observed only in insects and crustaceans—it nevertheless provides a glimpse into the complex immunoregulatory network orchestrated by SVWC proteins.

In the *Macrobrachium nipponense*, both *Mn*SVWC and *Mn*SVWC2 have been shown to possess PRR and signaling molecule functions, and notably, the same MnSVWC protein functions both as a signaling molecule for antiviral immunity and as a PRR for antimicrobial immunity [12,17,18]. In the silkworm *Bombyx mori*, BmSVWC also exhibits dual identity, but with a division of labor: *Bm*Vago (i.e., BmSVWC3) primarily acts as a signaling molecule for antiviral immunity [19], while BmSVWC2 functions primarily as a PRR for antimicrobial immunity [8]. Furthermore, in the *Litopenaeus vannamei*, the five *Lv*SVWC subtypes (*Lv*SVWC1-5) display distinct expression patterns and functional characteristics [5]; *Lv*SVWC1/4/5 significantly activate the JAK/STAT pathway, acting as cytokines for antiviral immunity, whereas other subtypes show a muted response to viral infection. In the *Penaeus monodon*, *Pm*Vago1, *Pm*Vago4, and *Pm*Vago5 respond strongly to white spot syndrome virus (WSSV) infection. However, RNA interference (RNAi) studies reveal that only knockdown of *Pm*Vago1 and *Pm*Vago4 significantly accelerates mortality and increases viral load. Moreover, the promoter activities of *Pm*Vago1 and *Pm*Vago4 are differentially regulated by the IKK-NF-κB pathway [20]. Although research on the functional differentiation of SVWCs in insects is not yet comprehensive, this evidence strongly suggests a hypothesis: have distinct SVWC subtypes evolved specific “professional divisions”—some dedicated to PRR functions, others to signaling, and yet others performing dual roles?

The diversity of SVWCs may enable them, much like vertebrate cytokines, to activate different JAK-STAT pathway variants through distinct receptor combinations, thereby initiating customized immune responses. Invertebrate genomes, while possessing fewer STAT genes (typically 1–3 compared to vertebrates), can generate multiple STAT variants through alternative splicing [21]. For instance, *Drosophila* has a single STAT gene (STAT92E), which produces up to 11 variants. *Bombyx mori* possesses at least two variants, STAT-L and STAT-S [22]. The *Aedes albopictus* contains six variants, with two variants already studied [23]. We further hypothesize that different SVWC subtypes may selectively activate specific STAT variants, thereby regulating distinct sets of effector genes—some subtypes dominating antiviral responses, others anti-Gram-negative bacterial responses, and others antifungal defenses—forming a finely divided immunoregulatory network.

While this hypothesis is appealing, direct evidence remains scarce. Currently, little is known about the specific pairing between SVWC subtypes and particular JAK/STAT variants. When acting as signaling molecules, are different subtypes induced by different upstream PRR pathways? Do they bind different integrin subtypes? Upon activation, do they result in distinct STAT dimer compositions and differential transcriptional outputs? As PRRs, do different SVWC subtypes specifically recognize particular microbes and subsequently activate distinct hemocyte phagocytosis or NF-κB pathways? Answers to these questions will determine whether the SVWC system can truly be termed an “invertebrate cytokine network.”

**Table 1:** Reported SVWC protein sequences from various invertebrates (The upper table is for insects, and the lower table is for crustaceans).

Species Name	Gene Name	Gene Accession Number	Response to Virus	References
*Aedes albopictus*	*Aa*Vago	XP_001658930	*Aa*Vago inhibits WNV replication	[2]
*Drosophila melanogaster*	*Dm*Vago	NP_001285106.1	DCV, FHV, SINV, and Nora virus upregulate *Dm*Vago expression	[24]
*Bombus terrestris*	*Bt*Vago	XP_003399812	IAPV and SBPV downregulate *Bt*Vago expression	[25]
*Culex quinquefasciatus*	*Cx*Vago	XP_001842264	*Cx*Vago inhibits WNV replication	[2,26]
*Musca domestica*	WHIS1	WBT97137.1	Bacteria upregulate WHIS1 expression	[27]
	*Md*SVWC	XP_005188181	*Md*SVWC inhibits bacteria	[28]
*Aedes aegypti*	*Ae*Vago1	XP_001658930.1	Wolbachia upregulates *Ae*Vago1 expression AeVago1 inhibits JCV and DENV replication	[29,30]
	*Ae*Vago2	XP_001658928	Wolbachia downregulates *Ae*Vago2 expression	[29]
*Bombyx mori*	*Bm*SVWC1	XP_012547804.1	Not reported	[31]
	*Bm*SVWC2	XP_004930780	*Bm*SVWC downregulates *Bm*NPV expression	[31]
	*Bm*Vago	XP_004928346.2	*Bm*Vago downregulates *Bm*NPV expression	[19,31]
*Eriocheir sinensis*	*Es*SVWC	WBW48830.1	*Es*SVWC inhibits bacteria	[32]
*Macrobrachium nipponense*	*Mn*SVWC	QCT05776.1	*Mn*SVWC inhibits WSSV and CMNV replication	[12,17]
	*Mn*SVWC2	XKT94494.1	WSSV and bacteria upregulate *Mn*SVWC2 expression	[18]
*Penaeus japonicus*	*Pj*Vago5	BAW78902	WSSV upregulates *Pj*Vago5 expression	[10]
	Vago-L	UBU90503.1	Vago-L inhibits WSSV replication	
*Litopenaeus vannamei*	*Lv*SVWC1	AEB54791.1	WSSV upregulates *Lv*SVWC1 expression	[5]
	*Lv*SVWC4	AEB54794.1	WSSV upregulates *Lv*SVWC4 expression	
	*Lv*SVWC5	AEB54795.1	WSSV upregulates *Lv*SVWC5 expression	
*Penaeus monodon*	*Pm*Vago1	WKV34892.1	*Pm*Vago1 inhibits WSSV replication	[20]
	*Pm*Vago4	WKV34895.1	*Pm*Vago4 inhibits WSSV replication	
	*Pm*Vago5	WKV34896.1	*Pm*Vago5 inhibits WSSV replication	

## 3. SVWC Activation and Signal Transduction: Multilayered Regulation of the JAK-STAT Pathway

Numerous SVWC subtypes exist, and reported protein sequences all contain NF-κB-like binding sites or IRF binding sites. SVWC protein activation is achieved through these conserved NF-κB binding sites in promoters or IRF binding sites in the 5′-untranslated region (5′-UTR). Some receptor proteins can even activate both pathways to induce expression of different SVWC subtypes [33].

### 3.1. Canonical NF-κB Pathways: Involvement of Dorsal, Rel-2, and Relish (Figure 4)

In invertebrates, nuclear factor kappa B (NF-κB) family members including Dorsal, Rel-2, and Relish can activate different SVWC subtypes. All these proteins possess a Rel homology domain (RHD), suggesting an intrinsic link between RHD and SVWC activation.

**Figure 4:**
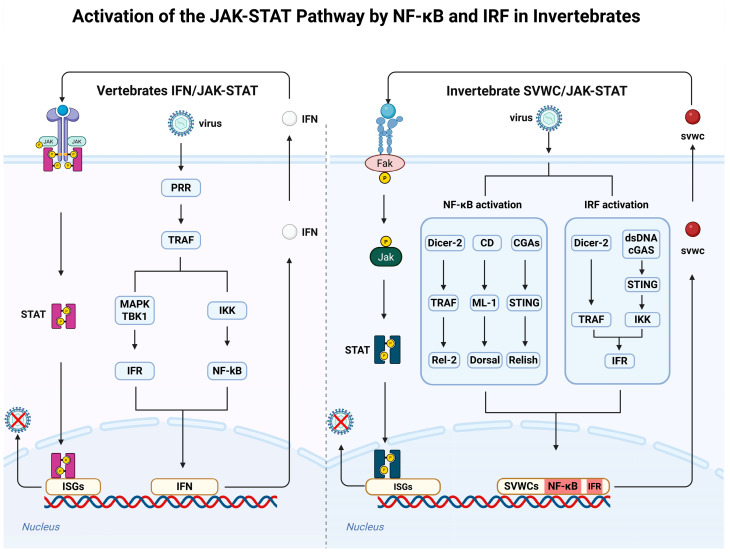
Invertebrate SVWC proteins can mediate antiviral immunity by activating the JAK/STAT pathway, exhibiting functional similarity to IFNs despite structural differences. SVWC activates the JAK/STAT pathway through both NF-κB (Dorsal, Rel-2, Relish) and IRF, forming a multi-pathway, precisely regulated network.

#### 3.1.1. Activation of SVWC by Rel-2

Dicer-2-TRAF-Rel-2 Axis: In *Culex* mosquitoes, following West Nile virus (WNV) infection, Dicer-2 activates the NF-κB family member Rel-2 via tumor necrosis factor receptor-associated factor (TRAF). Rel-2 then binds to conserved NF-κB sites in the *Cx*SVWC promoter to drive its expression [26]. Notably, Dicer-2-mediated activation of SVWC is highly conserved; Dicer-2 from *Drosophila* and *Litopenaeus vannamei* can mutually activate each other’s SVWC promoters [5].

#### 3.1.2. Activation of SVWC by Dorsal

CD–ML1–Dorsal Axis: In the *Marsupenaeus japonicus*, the myeloid differentiation factor 2 (MD-2)-related lipid-recognition (ML) domain directly recognizes the envelope lipid component cholesta-3,5-diene (CD) of white spot syndrome virus (WSSV). This recognition induces nuclear translocation of Dorsal, leading to SVWC expression [34].

Furthermore, studies in *Litopenaeus vannamei* indicate that Dorsal activation is also associated with meiotic recombination 11 (MRE11), a cytosolic sensor of double-stranded DNA (dsDNA). MRE11 can activate Dorsal to regulate antiviral immunity, hinting at a potential MRE11-STING-IRF axis [33].

#### 3.1.3. Activation of SVWC by Relish

cGAS-*Bm*STING-*Bm*Relish Axis: Upon infection with *Bombyx mori* nucleopolyhedrovirus (BmNPV), the DNA sensor cyclic GMP-AMP synthase (cGAS) activates the stimulator of interferon genes (STING). STING subsequently activates Relish, inducing *Bm*SVWC expression [19]. Additionally, in shrimp, STING can also act as a PRR directly binding viral dsDNA [35].

### 3.2. Activation of SVWC by IRF (Figure 4)

#### 3.2.1. Dicer-2-TRAF-IRF Axis

In *Litopenaeus vannamei*, Dicer-2 activates *Lv*IRF via TRAF3. *Lv*IRF then binds to the 5’-UTR of *Lv*Vago4 and *Lv*Vago5 to activate their transcription [36]. Notably, the 5′-UTR of the *Lv*IRF gene contains a simple sequence repeat (SSR). Shorter repeat lengths of (CT)_n_ correlate with higher *Lv*IRF expression and increased viral tolerance [37], implying that genetic polymorphisms may influence the induction efficiency of specific SVWC subtypes.

#### 3.2.2. dsDNA/cGAS → STING → IKKε → IRF Axis

In *Litopenaeus vannamei*, the STING homolog can directly recognize cGAS or viral DNA, inducing *Lv*Vago4 expression and establishing an antiviral state and initiating an interferon-like antiviral response [35].

This pathway has also been validated in *Penaeus monodon*: overexpression of *Pm*IKK activates the promoter activity of *Pm*Vago1 and *Pm*Vago4, and these two subtypes can further activate the promoters of Dorsal and Relish [20]. Additionally, meiotic recombination protein 11 (MRE11) in *Litopenaeus vannamei* can activate the STING-IRF axis, regulating IRF-mediated antiviral responses [33].

### 3.3. Unique Signal Transduction: SVWC Activates JAK-STAT via Integrins

The mechanism by which SVWCs, acting as cytokines, activate the JAK-STAT pathway has long been enigmatic. Mammalian JAK-STAT activation typically requires ligand binding to receptors and induction of receptor dimerization [38]. As Domeless (Dome) is the sole Jak/Stat transmembrane receptor in invertebrate genomes [39], it was initially hypothesized that Dome might be recognized by a ligand to activate the JAK/STAT pathway. However, studies in *Culex* mosquitoes [2] and kuruma shrimp [10] have shown that Dome knockdown does not affect Vago-mediated JAK-STAT activation, suggesting a non-canonical mechanism for SVWC-mediated JAK-STAT activation in invertebrates.

Crucial evidence comes from *Bombyx mori*: downstream of the *Bm*STING-*Bm*Relish signaling pathway, *Bm*SVWC directly interacts with *Bm*integrin β1. Inhibition of integrin β1 blocks JAK-STAT activation [19]. Direct confirmation was provided in Marsupenaeus japonicus: *Mj*Vago-L directly interacts with the integrin β3 subunit, and an aspartic acid residue (Asp) in *Mj*Vago-L is essential for this interaction and its antiviral function. Furthermore, the key integrin signaling adaptor protein focal adhesion kinase (Fak) mediates *Mj*Vago-L activation of the Jak/Stat pathway, forming the *Mj*Vago-L–Integrin–Fak–Jak–Stat–Ficolin signaling axis [25].

Although the presence of integrin recognition motifs (e.g., Arg-Gly-Asp [RGD]/Lys-Gly-Asp [KGD]) in *Drosophila* and *Culex* Vago suggests that the Vago-L/Integrin/Jak/Stat signaling axis may be widespread in arthropod antiviral immunity [25], there is currently no evidence confirming whether the *Mj*Vago-L/integrin-mediated Jak/Stat pathway is conserved across arthropods.

This mechanism links cytokine signaling with the integrin pathway. Crucially, integrins themselves constitute a diverse receptor family—different α and β subunit combinations form various integrin heterodimers. This raises an intriguing hypothesis: different SVWC subtypes may selectively bind different integrin heterodimers, thereby activating distinct intracellular signaling pathways (such as different JAK variants) and ultimately initiating different transcriptional programs. The conservation of integrin recognition motifs in *Drosophila* and *Culex* Vago suggests this mechanism is conserved in insects. However, the “pairing rules” between SVWC subtypes and integrin subtypes remain an open question.

## 4. Emerging Functions of SVWCs: Acting as PRRs in Antimicrobial and Antiviral Immunity

**Figure 5:**
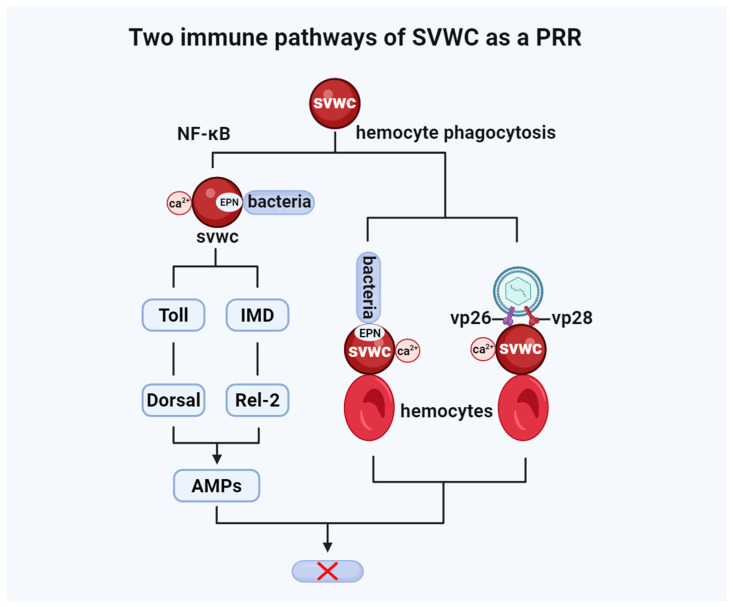
SVWC proteins, acting as pattern recognition receptors (PRRs), can promote the expression of antimicrobial peptides (AMPs) and other effectors by activating the NF-κB (Toll and IMD) pathways to mediate antibacterial immunity. Additionally, they can enhance antiviral and antibacterial immune responses by facilitating hemocyte phagocytosis.

The role of SVWCs extends beyond that of a cytokine and their function is not limited to antiviral immunity. Studies in crustaceans have revealed that SVWC proteins not only act as IFN analogs activating the JAK-STAT pathway for antiviral responses but also function as PRRs upon infection with viruses or bacteria. In this capacity, they trigger NF-κB (Toll and IMD) pathways, exerting immune effects against bacteria and viruses (Figure 5) [11].

### 4.1. Regulators of Humoral Immunity: Activating Toll and IMD Pathways

As PRRs, SVWC proteins can specifically bind polysaccharide PAMPs from bacteria and fungi, activating the Toll and immune deficiency (IMD) signaling pathways (which primarily recognize Gram-positive bacteria/fungi and Gram-negative bacteria, respectively [24]. These pathways regulate the expression of immune-related genes, including antimicrobial peptides (AMPs), via the NF-κB transcription factors Dorsal and Relish. This binding process is Ca^2+^-dependent and involves specific sugar recognition, indicating calcium-dependent lectin activity. For example, in *Bombyx mori*, high expression of BmSVWC2 enhances pathogen recognition, signal transduction, and effector molecule production within the Toll and IMD pathways, inducing the production of various AMPs and conferring significant antimicrobial activity to the hemolymph [8]. Similarly, *Musca domestica Md*SVWC1 [28] and *Eriocheir sinensis Es*SVWC [32] can act as PRRs specifically recognizing bacterial PAMPs and activating Toll/IMD pathways for antibacterial immunity. Notably, in *Macrobrachium nipponense*, the recognition of microbial PAMPs (such as lipopolysaccharide [LPS] or Lys-type peptidoglycan) by SVWC acting as a PRR may depend on a glutamate–proline–asparagine (Glu-Pro-Asn, EPN) motif. Mutation of this EPN motif in SVWC proteins reduces their affinity for PAMPs from various microbes (e.g., *Escherichia coli*, *Staphylococcus aureus*, and *Pichia pastoris*) [17].

### 4.2. Promoters of Cellular Immunity: Mediating Hemocyte Phagocytosis

In *Macrobrachium nipponense*, co-incubation of recombinant *rMn*SVWC2 with *E. coli* prior to injection significantly enhances hemocyte phagocytosis of invading bacteria in vivo [18], a process dependent on the Ca^2+^ molecular switch. SVWCs, as secreted proteins, can agglutinate invading microbes and viruses and promote hemocyte phagocytosis [32]. They circulate in the hemolymph, binding PAMPs (like LPS or Lys-type peptidoglycan) on one end [17,18] and hemocyte surfaces on the other, acting as “molecular bridges” to facilitate phagocytosis. Furthermore, *Bm*SVWC2 [8] and *Md*SVWC1 [28] also promote microbial phagocytosis by hemocytes.

Similarly, in *Macrobrachium nipponense*, recombinant *Mn*SVWC coated onto WSSV enhances hemocyte phagocytosis of the virus, promoting viral clearance. *Mn*SVWC recognizes WSSV by binding the envelope proteins VP26 and VP28, a recognition process dependent on calmodulin [12]. Although later-identified *Mn*SVWC2 binds only VP26 and not VP28 [18], this does not preclude the possibility of an “envelope protein-SVWC-calmodulin-clathrin”-dependent hemocyte phagocytosis mechanism regulating WSSV clearance in insects.

It is noteworthy that SVWC functions vary across species. *Mn*SVWC mediates hemocyte phagocytosis of viruses and microbes but does not appear to block the NF-κB pathway affecting AMP expression [17]. *Es*SVWC has only been found to regulate AMPs for antimicrobial immunity, whereas *Bm*SVWC and *Md*SVWC can mediate antimicrobial immunity via both cellular (phagocytosis) and humoral (AMPs) pathways. This raises the question: Are different pathways and functions executed by different SVWC subtypes? Is there functional overlap and division of labor among subtypes?

Intriguingly, Wolbachia endosymbionts can inhibit viral replication. In *Aedes aegypti*, *Wolbachia* infection effectively reduces the replication of Jamestown Canyon virus (JCV) [30] and dengue virus (DENV) [29], with the inhibition of JCV occurring primarily during early infection. This phenomenon is thought to be linked to SVWC proteins. Studies on DENV infection in *Aedes aegypti* indicate that *Wolbachia* infection significantly upregulates *Ae*Vago1 expression, thereby inhibiting viral replication. In JCV-infected *Aedes aegypti*, *Wolbachia* similarly upregulates *Ae*Vago1, particularly during the early stages of *Wolbachia* infection, which coincides with the early-stage inhibition of JCV [30]. This supports the notion that *Wolbachia* inhibits JCV and DENV replication by activating Vago expression. This finding has significant positive implications for controlling viruses transmitted to humans by vectors like *Aedes aegypti*, contributing to population safety [40]. An interesting question arises: Why is *Ae*Vago1 in *Aedes aegypti* not directly induced by DENV infection, yet its induction by *Wolbachia* still exerts an antiviral effect against DENV?

## 5. Conclusions and Perspectives: SVWC—Potentially Constructing a Diversified Cytokine Network in Invertebrate Immunity

Insects hold significant economic and nutritional value, encompassing well-known species like honeybees [41,42,43,44,45,46] and silkworms [47,48,49], as well as mealworms [50,51] and various edible insects [52]. SVWC proteins have high translational potential for enhancing disease resistance in economically important insects and crustaceans.

In-depth study of the SVWC protein family has reshaped our understanding of innate immunity in insects and crustaceans. Since the initial identification of Drosophila Vago in 2004, the functional perception of this protein has evolved from an “interferon analog” to a “multifunctional immune hub.” Current evidence indicates that SVWC is a highly diverse protein family, and its subtype diversity represents a strategy for functional specialization. Different SVWC subtypes exhibit distinct downstream activation targets and upstream induction pathways. Some act primarily as PRRs directly recognizing pathogens, others function as cytokine-like signaling molecules activating the JAK-STAT pathway, and some possess dual functionality. SVWC expression is under the precise control of both NF-κB (Dorsal, Rel-2, Relish) and IRF pathways. Different pathogen sensors (Dicer-2, cGAS-STING, MRE11, etc.) induce the expression of specific SVWC subtypes via particular transcription factors, establishing specific “pathogen type-induction pathway-SVWC subtype” pairings. Importantly, evidence reveals a unique mechanism by which SVWCs activate the JAK-STAT pathway via integrins rather than the canonical Domeless receptor, marking the first link between cytokine signaling and the integrin pathway. Given the indispensable importance of external microbes for insect environmental adaptation, the impact of SVWCs on insect microbial immunity is equally crucial [53]. As PRRs, SVWCs not only activate Toll and IMD pathways to induce AMP expression but also agglutinate pathogens and promote hemocyte phagocytosis, extending their function from antiviral to antibacterial immunity and from humoral to cellular immunity. Based on these findings, we propose that the SVWC family potentially constitutes a multilayered immunoregulatory network: diverse SVWC subtypes are induced by distinct upstream pathways, recognize different pathogens, bind different integrin receptors, activate different STAT variants, and ultimately initiate tailored transcriptional programs.

While the “diversified cytokine network” model for SVWC proteins is compelling, significant gaps remain. First, our understanding of all SVWC subtypes’ functions is incomplete. Current research is limited to a few insect and crustacean species and is fragmented across viral and microbial immunity studies. Evidence supporting the proposed pathway mechanisms is not yet abundant. Direct evidence for SVWC activating the JAK/STAT pathway via integrins, independent of the classical Domeless receptor, exists only in *Bombyx mori* and *Marsupenaeus japonicus*. Indirect evidence is limited to *Marsupenaeus japonicus* and *Culex* mosquitoes. Although integrin recognition motifs in *Drosophila* and *Culex* Vago suggest that integrin-mediated Jak/Stat activation might be widespread in arthropods, sufficient evidence confirming the conservation of this pathway across arthropods is currently lacking. Specifically, direct evidence supporting the hypothesis that different subtypes specifically activate corresponding downstream STATs is scarce. These shortcomings require further exploration over time.

Second, while current findings generally support the hypothesis of subtype-specific SVWC immunity against microbes and viruses, the observation that *Wolbachia* activates *Ae*Vago expression while indirectly inhibiting viral replication raises questions. Does this imply that SVWC-mediated immunity against viruses and microbes is compatible or can occur simultaneously? Furthermore, in *Penaeus monodon*, *Pm*Vago1, *Pm*Vago4, and *Pm*Vago5 all exhibit high responsiveness to WSSV infection, yet only the knockdown of *Pm*Vago1 and *Pm*Vago4 leads to increased viral load. In *Wolbachia*-infected *Aedes aegypti*, *Ae*Vago1 is upregulated while *Ae*Vago2 is downregulated. This prompts questions: Do synergistic or antagonistic relationships exist among different SVWC subtypes? These issues remain largely unexplored. Although the SVWC family has profoundly changed our understanding of cytokine-like signaling pathways in arthropods, significant knowledge gaps remain in this field. Addressing these questions will refine the theoretical foundation of invertebrate immunology.

In conclusion, ongoing research into the SVWC protein family is fundamentally reshaping our view of insect innate immunity. Their existence and diversification stand as vivid testament to life’s evolutionary arms race over billions of years—a process of continuous optimization, integration, and innovation of defense strategies. Invertebrates, it turns out, also possess a complex, sophisticated, and diversified “cytokine-like” immunoregulatory network.

## Data Availability

No new data were created or analyzed in this study. Data sharing is not applicable to this article.
